# Genome-wide identification and comprehensive characterization of the *Aux/IAA* gene family in *Cucurbita moschata* and its responses analysis to abiotic stresses

**DOI:** 10.1186/s12870-026-09733-x

**Published:** 2026-08-13

**Authors:** Mengyao Sun, Jiayi Duan, Lina Jiang, Junguo Zhou, Yufei Zhai

**Affiliations:** Henan Institute of Science and Technology, Xinxiang, Henan 453000 P. R. China

**Keywords:** *Cucurbita moschata*, Phylogenetic analysis, Cis-regulatory elements, Abiotic stresses

## Abstract

**Background:**

Auxin is a central phytohormone involved in regulating plant growth, development, and stress responses, with the *Aux/IAA* gene family functioning as an essential component of the auxin signaling pathway. To elucidate the genomic features and potential functions of the *Aux/IAA* gene family in pumpkin (*Cucurbita moschata*), we performed a genome-wide identification and systematic characterization.

**Results:**

A total of 72 *CmIAA* genes were identified, encoding proteins ranging from 158 to 1275 amino acids with predicted isoelectric points of 4.57–9.81. These genes were unevenly distributed across 20 chromosomes, with Chr17 harboring the highest number, while no *CmIAA* genes were detected on Chr3. Phylogenetic analysis classified the genes into nine subgroups (Groups Ⅰ–Ⅸ), with Groups Ⅰ, Ⅵ, and Ⅸ exhibiting relative expansion. Gene structure and conserved motif analyses revealed subgroup-specific motif compositions, with motif 1 representing the core conserved domain. Intraspecific collinearity analysis identified 54 segmentally duplicated gene pairs but no tandem duplication events, whereas interspecific synteny revealed extensive orthologous relationships between pumpkin and *Cucurbita pepo* and *Cucurbita maxima*. Promoter analysis showed that *CmIAA* genes contain multiple cis-elements associated with hormone responses and abiotic stress responses, including ABRE, MBS, and DRE. Tissue expression analysis demonstrated that many *CmIAA* genes exhibited tissue-preferential expression patterns. Under abiotic stress conditions, *CmIAA*69 showed a salt-specific expression pattern, whereas *CmIAA*39 and *CmIAA*58 responded to both salt and drought treatments, indicating that these *CmIAA* genes play distinct roles in pumpkin responses to different abiotic stresses.

**Conclusions:**

This study systematically characterized the *Aux/IAA* gene family in pumpkin, highlighting its evolutionary diversity, structural conservation, and distinct regulatory features. These findings provide valuable genetic resources for further functional studies and inform the potential roles of *CmIAA* genes in abiotic stress responses.

**Supplementary Information:**

The online version contains supplementary material available at https://doi.org/10.1186/s12870-026-09733-x.

## Background

Auxin (indole-3-acetic acid) is a central phytohormone that regulates diverse aspects of plant growth, development, and environmental responses [1, 2]. The biological activity of auxin is tightly controlled by multiple regulatory processes, including auxin biosynthesis, transport, metabolism, and signal transduction, which together determine cellular auxin availability and downstream responses [3]. The biological effects of auxin are mainly mediated through the conserved TIR1/AFB-Aux/IAA-ARF signaling pathway, which serves as a key regulatory module connecting auxin perception with downstream transcriptional responses [4]. Aux/IAA proteins are short-lived, nucleus-localized transcriptional repressors that interact with auxin response factors (ARFs) and inhibit the expression of auxin-responsive genes under low auxin conditions [5, 6]. When auxin accumulates, Aux/IAA proteins are recognized by the SCF^TIR1/AFB ubiquitin ligase complex and rapidly degraded through the ubiquitin-proteasome pathway, thereby releasing ARFs to activate downstream auxin-responsive genes [7]. This dynamic regulatory mechanism enables plants to precisely control developmental processes and environmental responses through modulation of auxin signaling outputs. Recent studies have further demonstrated that the TIR1/AFB-Aux/IAA-ARF module plays a central role in regulating cell expansion and growth-related processes [8, 9], highlighting the importance of Aux/IAA proteins as key regulators in auxin-mediated biological functions.

As essential components of auxin signaling, Aux/IAA proteins constitute a multigene family that has been identified in numerous plant species. Genome-wide analyses have revealed considerable variation in Aux/IAA family size among different species, including 89 members identified in turnip [1], 119 in *Brassica napus* L [10], 36 in *Hordeum vulgare *[11], 31 in *Oryza sativa* [12], and 25 in *Solanum lycopersicum* [13]. The expansion of *Aux/IAA* gene families is mainly attributed to genome duplication events, including whole-genome duplication and segmental duplication, followed by evolutionary divergence that contributes to functional specialization among family members [4, 14]. Due to their diverse expression patterns and regulatory characteristics, different Aux/IAA members have been shown to participate in distinct developmental processes and environmental responses. Therefore, systematic characterization of *Aux/IAA* gene families provides an effective approach for understanding the evolutionary diversification and potential biological functions of auxin signaling components.

Beyond their roles in plant growth and development, increasing evidence indicates that *Aux/IAA* genes are involved in responses to various abiotic stresses by integrating hormonal signaling with environmental cues [15, 16]. Drought and salinity are among the most severe environmental constraints limiting crop productivity worldwide, as they disrupt water homeostasis, cause ion imbalance, and induce oxidative damage, ultimately affecting plant growth and yield formation [17]. Increasing evidence suggests that auxin signaling participates in stress regulation through modulation of root development, hormone interactions, and stress-responsive gene expression [18]. In *Capsicum annuum*, numerous *Aux/IAA* genes exhibit strong induction under salt and drought stress [19]; in *Solanum lycopersicum*, several members show time-dependent expression following stress exposure [20]; while overexpression of *OsIAA*18 significantly enhances drought and salt tolerance in *Oryza sativa* [21, 22]. Stress-responsive expression patterns of *Aux/IAA* genes have been observed in several plant species, including *Zea mays*, *Glycine max*, and *Cicer arietinum* [23, 24]. These findings indicate that *Aux/IAA* genes serve as important regulatory components linking auxin signaling with plant responses to adverse environmental conditions [25–27].

Pumpkin (*Cucurbita moschata*), an economically important crop belonging to the Cucurbitaceae family, is widely cultivated due to its nutritional value and strong environmental adaptability. In addition, pumpkin possesses a vigorous root system and excellent stress tolerance, making it an important rootstock for grafting crops such as cucumber and watermelon to enhance resistance against environmental stresses [28, 29]. Given the widespread occurrence of soil secondary salinization and frequent drought events in protected cultivation systems, improving the stress tolerance of pumpkin rootstocks has become a critical factor for ensuring the sustainable production of grafted vegetable crops. However, pumpkin production is frequently challenged by abiotic stresses, particularly drought and salinity, which negatively affect plant growth, development, yield, and quality [30, 31]. Although *Aux/IAA* gene families have been systematically characterized in several plant species, including tomato, spinach, and potato [32, 33], the evolutionary characteristics, regulatory features, and stress-responsive expression patterns of *Aux/IAA* genes in pumpkin remain largely unknown. Therefore, comprehensive identification and characterization of the Aux/IAA gene family in pumpkin are necessary to reveal their potential roles in auxin-mediated growth regulation and abiotic stress responses.

In this study, we performed a genome-wide identification and systematic analysis of the *Aux/IAA* gene family in pumpkin, including phylogenetic relationships, gene structures, conserved motifs, duplication patterns, cis-regulatory elements, tissue-specific expression profiles, and responses to salt and drought stresses. This study provides a comprehensive overview of the CmIAA family and identifies candidate genes for further functional studies of Aux/IAA-mediated regulation in pumpkin growth and abiotic stress responses.

## Results

### Identification of gene family members and analysis of physicochemical properties

A genome-wide search was conducted, and a total of 72 *Aux/IAA* genes were identified in *Cucurbita moschata.* These genes were designated as *CmIAA*1–*CmIAA*72 according to their chromosomal positions (Table 1). The encoded CmIAA proteins exhibited considerable diversity in their physicochemical properties. The protein lengths ranged from 158 to 1275 amino acids, with predicted molecular weights ranging from 17.79 to 141.56 kDa. The theoretical isoelectric points (pI) varied from 4.57 to 9.81, indicating differences in the biochemical characteristics among CmIAA proteins.

**Table 1:** Informantion of *Aux/IAA* genes identified in *C. moschata*

Genome Name	Gene ID	Chromosome location	Amino Acids Number（aa)	Isoelecttic point (pI)	Molecular weight (Da)
CmIAA1	CmoCh01G000950.1	Chr01	315	8.56	34523.77
CmIAA2	CmoCh01G001250.1	Chr01	196	7.6	21943.88
CmIAA3	CmoCh01G001260.1	Chr01	158	8.86	17788.55
CmIAA4	CmoCh01G014970.1	Chr01	989	6.83	111111.61
CmIAA5	CmoCh01G015610.1	Chr01	1160	6.46	129445.48
CmIAA6	CmoCh01G020170.1	Chr01	967	5.3	106455.11
CmIAA7	CmoCh02G008800.1	Chr02	795	6.44	87897.75
CmIAA8	CmoCh02G009300.1	Chr02	734	5.84	82159.66
CmIAA9	CmoCh02G017600.1	Chr02	279	6.98	30257.8
CmIAA10	CmoCh02G018170.1	Chr02	1275	6.62	141556.39
CmIAA11	CmoCh04G007210.1	Chr04	709	6.68	79522.43
CmIAA12	CmoCh04G015610.1	Chr04	199	4.57	22700.13
CmIAA13	CmoCh04G020040.1	Chr04	754	6.67	83790.74
CmIAA14	CmoCh04G023590.1	Chr04	1151	6.24	128435.87
CmIAA15	CmoCh04G028890.1	Chr04	856	6.08	94971.03
CmIAA16	CmoCh05G003790.1	Chr05	190	5.81	21935.77
CmIAA17	CmoCh05G005900.1	Chr05	363	6.57	39872.95
CmIAA18	CmoCh05G007670.1	Chr05	1114	6.71	122841.22
CmIAA19	CmoCh05G012250.1	Chr05	219	9.81	24420.31
CmIAA20	CmoCh05G012260.1	Chr05	238	5.34	26073.3
CmIAA21	CmoCh05G014250.1	Chr05	1120	5.99	124417.52
CmIAA22	CmoCh06G000030.1	Chr06	445	5.74	49299.65
CmIAA23	CmoCh06G009480.1	Chr06	180	8.91	19972.77
CmIAA24	CmoCh06G009490.1	Chr06	242	7.51	26404.25
CmIAA25	CmoCh06G013550.1	Chr06	232	6.41	25180.78
CmIAA26	CmoCh06G013570.1	Chr06	198	5.2	21708.52
CmIAA27	CmoCh07G000800.1	Chr07	992	6.46	110843.78
CmIAA28	CmoCh07G006010.1	Chr07	371	6.11	40607.65
CmIAA29	CmoCh08G001220.1	Chr08	748	6.2	83770.35
CmIAA30	CmoCh08G003010.1	Chr08	201	5.35	22316.16
CmIAA31	CmoCh08G003030.1	Chr08	284	8.53	31195.46
CmIAA32	CmoCh08G006140.1	Chr08	182	9.11	20576.65
CmIAA33	CmoCh08G010880.1	Chr08	252	8.93	27492.62
CmIAA34	CmoCh08G010900.1	Chr08	179	8.78	19631.3
CmIAA35	CmoCh09G001150.1	Chr09	912	5.27	100533.64
CmIAA36	CmoCh09G004620.1	Chr09	237	5.3	27200.51
CmIAA37	CmoCh09G006100.1	Chr09	352	7.13	39097.26
CmIAA38	CmoCh10G000150.1	Chr10	849	5.74	93598.16
CmIAA39	CmoCh10G005700.1	Chr10	217	8.51	24928.24
CmIAA40	CmoCh10G006330.1	Chr10	198	5.01	22276.18
CmIAA41	CmoCh10G006720.1	Chr10	306	5.93	32976.92
CmIAA42	CmoCh11G005280.1	Chr11	207	5.98	23780.77
CmIAA43	CmoCh11G005910.1	Chr11	202	7.72	22947.97
CmIAA44	CmoCh11G006340.1	Chr11	317	5.62	34228.7
CmIAA45	CmoCh11G009990.1	Chr11	225	8.85	24684.21
CmIAA46	CmoCh12G001340.1	Chr12	355	5.69	39109.83
CmIAA47	CmoCh12G008890.1	Chr12	167	4.94	18641.75
CmIAA48	CmoCh12G009300.1	Chr12	1115	6.3	123145.44
CmIAA49	CmoCh12G011430.1	Chr12	195	5.41	21603.61
CmIAA50	CmoCh12G013660.1	Chr12	1178	6.15	131480.24
CmIAA51	CmoCh13G005400.1	Chr13	1000	5.76	111348.32
CmIAA52	CmoCh14G013230.1	Chr14	313	8.08	34006.39
CmIAA53	CmoCh14G013540.1	Chr14	173	9.14	19626.22
CmIAA54	CmoCh14G018090.1	Chr14	204	5.64	22628.43
CmIAA55	CmoCh14G018110.1	Chr14	241	6.41	26181.88
CmIAA56	CmoCh14G018910.1	Chr14	313	7.79	33071.26
CmIAA57	CmoCh15G008070.1	Chr15	853	5.83	95201.03
CmIAA58	CmoCh15G011500.1	Chr15	241	6.6	27733.81
CmIAA59	CmoCh15G013610.1	Chr15	695	6.05	77187.35
CmIAA60	CmoCh16G005850.1	Chr16	694	7.09	77641.58
CmIAA61	CmoCh17G001350.1	Chr17	296	7.55	31659.66
CmIAA62	CmoCh17G001860.1	Chr17	250	7.52	26877.73
CmIAA63	CmoCh17G001880.1	Chr17	195	5.67	21693.74
CmIAA64	CmoCh17G007560.1	Chr17	343	8.46	37198.99
CmIAA65	CmoCh17G007960.1	Chr17	183	5.57	20683.4
CmIAA66	CmoCh17G011930.1	Chr17	195	8.68	21874.74
CmIAA67	CmoCh17G011940.1	Chr17	234	7.59	25779.33
CmIAA68	CmoCh18G004680.1	Chr18	903	6.01	99859.28
CmIAA69	CmoCh18G010050.1	Chr18	182	5.83	21052.92
CmIAA70	CmoCh19G000550.1	Chr19	202	9.62	23005.49
CmIAA71	CmoCh19G001290.1	Chr19	281	7.71	30112.73
CmIAA72	CmoCh20G005090.1	Chr20	779	6.38	86292.74

The identified CmIAA proteins showed variation in size and physicochemical properties, suggesting that members of this family possess diverse molecular characteristics. These differences provide basic information for further classification and functional characterisation of individual CmIAA members.

### Chromosomal localization of the *CmIAA* gene family

**Fig. 1:**
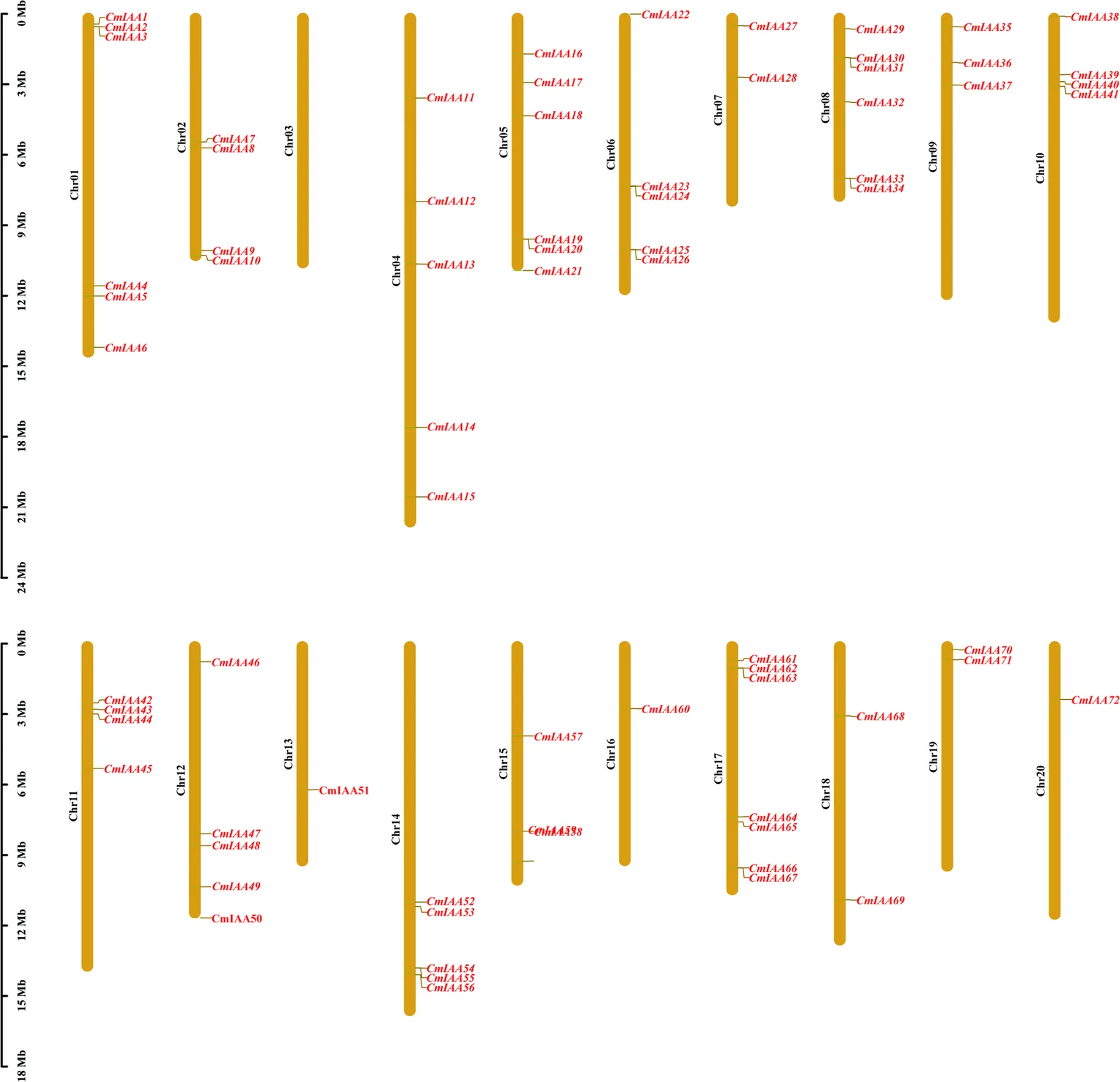
Chromosomal distribution of *CmIAA* genes in *C. moschata*

Chromosomal localization analysis revealed that the 72 *CmIAA* genes were unevenly distributed across the 20 chromosomes of the pumpkin genome (Fig. 1). The number of *CmIAA* genes on each chromosome ranged from zero to seven. Chromosome (Chr) 17 contained the highest number of *CmIAA* genes (seven), followed by Chr1, Chr5, and Chr8, each carrying six *CmIAA* genes. Chr4, Chr6, Chr12, and Chr14contained five *CmIAA* genes each. In contrast, only one *CmIAA* gene was identified on Chr13, Chr16, and Chr20, and no *CmIAA* gene was detected on Chr3.

Although Chr4 represents the longest chromosome in the pumpkin genome, it contained only five *CmIAA* genes, indicating that chromosome length alone does not determine *CmIAA* gene distribution. In addition, a small cluster containing three *CmIAA* genes was observed on Chr1, suggesting a localised accumulation pattern of some *CmIAA* members.

### Phylogenetic analysis of the *CmIAA* gene family

**Fig. 2:**
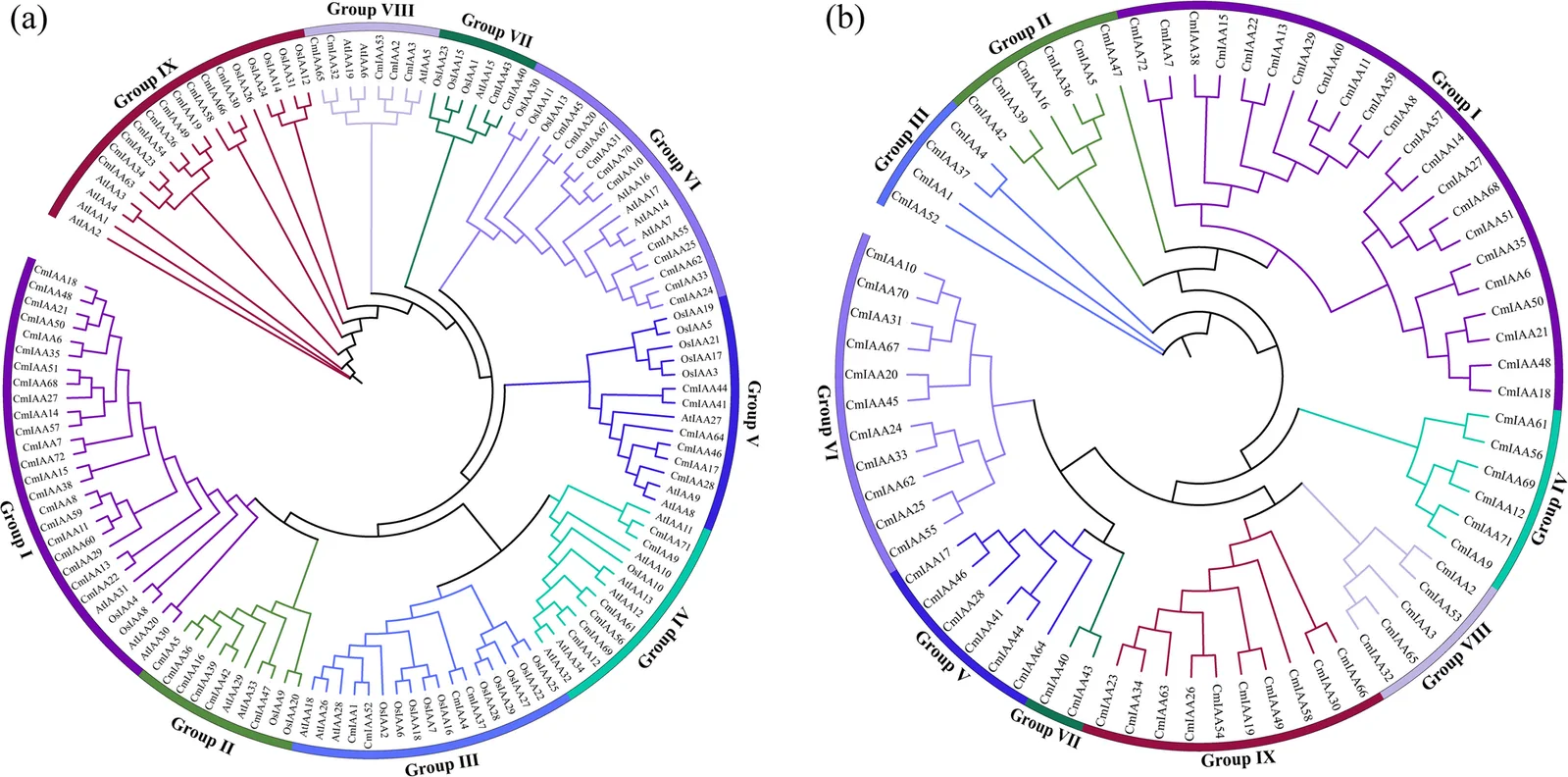
Phylogenetic analysis of Aux/IAA proteins. **a **Phylogenetic relationships of Aux/IAA proteins from *Arabidopsis thaliana*, *Oryza sativa*, and *C. moschata*. **b **Phylogenetic relationships of Aux/IAA proteins in *C. moschata*. The phylogenetic trees were constructed using the neighbor-joining (NJ) method implemented in MEGA-X software. Bootstrap analysis was performed with 1000 replicates to assess branch reliability. The nine subgroups (Group I-IX) are indicated by different colored sectors

To investigate the evolutionary relationships of the Aux/IAA family, a neighbour–joining (NJ) phylogenetic tree was constructed using 132 Aux/IAA protein sequences from pumpkin, *Arabidopsis thaliana*, and *Oryza sativa*. The phylogenetic analysis classified these proteins into nine conserved groups (Groups Ⅰ to Ⅸ) in Fig. 2a. The 72 CmIAA proteins were distributed across all nine groups, indicating that pumpkin Aux/IAA members shared conserved evolutionary relationships with Aux/IAA proteins from both dicot and monocot species. Among these groups, Groups Ⅰ, Ⅵ, and Ⅸ contained relatively more CmIAA members, whereas other groups showed fewer members.

The phylogenetic distribution also revealed differences in clustering patterns among species. In Groups V, Ⅵ, and Ⅸ, CmIAA proteins showed closer clustering relationships with *Arabidopsis thaliana* Aux/IAA proteins, while in Group VII, several CmIAA proteins clustered more closely with *Oryza sativa* members (Fig. 2a). These results reflect the evolutionary relationships of Aux/IAA proteins among different plant lineages.

To further characterise the evolutionary relationships within the pumpkin Aux/IAA family, a separate phylogenetic tree was constructed using the 72 CmIAA protein sequences (Fig. 2b). Consistent with the inter-species phylogenetic analysis, CmIAA proteins were classified into the same nine groups (Groups Ⅰ–Ⅸ). The number of members varied among different groups, with Groups V, Ⅵ, and Ⅸ containing larger numbers of CmIAA proteins. Members within the same subgroup were closely clustered with relatively short branch lengths, indicating high sequence similarity among these proteins.

### Conserved motif distribution and gene structure analysis of *CmIAA* genes

**Fig. 3:**
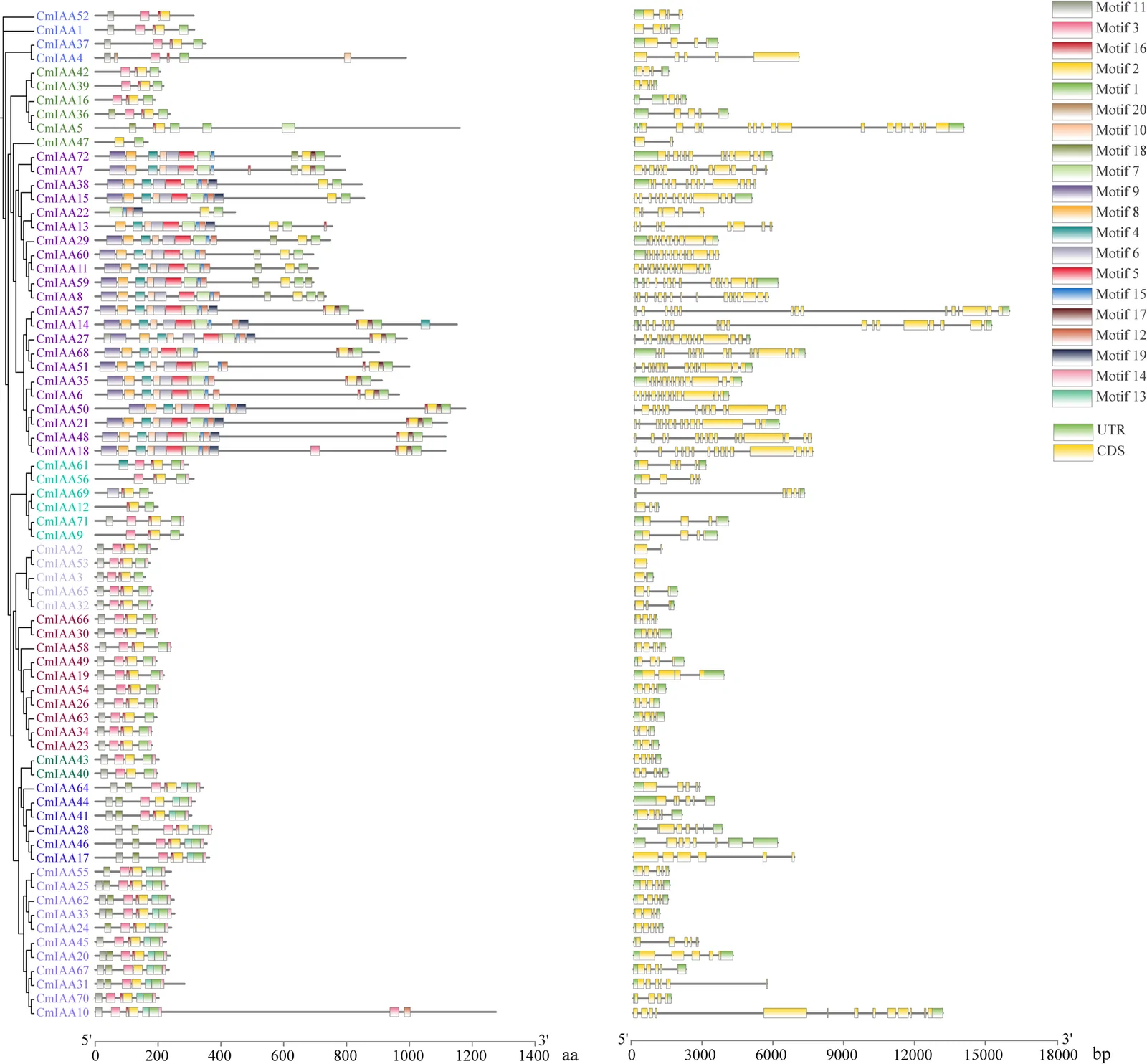
Phylogenetic grouping, conserved motif composition, and gene structure of *CmIAA* genes in *C. moschata*. **a **The panel shows the conserved motifs of CmIAA proteins identified by MEME, with different colors representing distinct motifs. **b **The panel illustrates the exon-intron structures of *CmIAA* genes, where yellow boxes indicate coding sequences (CDSs), green boxes represent untranslated regions (UTRs), and black lines denote introns. All *CmIAA* genes are arranged according to their positions in the protein-based phylogenetic tree. (The left scale indicates protein length in amino acids (aa), whereas the right scale represents gene length in base pairs (bp) )

To characterise the structural features of the *CmIAA* gene family, conserved motifs and exon-intron structures were analysed in relation to the phylogenetic classification (Fig. 3). A total of 20 conserved motifs were identified among the 72 CmIAA proteins. Motif 1 was present in most CmIAA proteins, indicating its widespread conservation among Aux/IAA members. Proteins belonging to the same phylogenetic subgroup generally exhibited similar motif compositions and arrangements, consistent with their phylogenetic relationships. In addition to conserved motifs shared among different groups, several subgroup-specific motifs were identified. For example, motifs 4, 5, 9, 15, and 19 were specifically detected in Group Ⅰ, whereas motif 13 was mainly present in Groups Ⅴ and Ⅵ. Conversely, motifs 3 and 11 were widely distributed among multiple subgroups but were absent from Group Ⅰ. These differences in motif composition revealed structural variation among different CmIAA subgroups.

The exon-intron organisation of *CmIAA* genes was further analysed to assess structural characteristics. Most *CmIAA* genes contained two to four exons, with similar exon numbers and arrangements observed among members within the same phylogenetic subgroup. However, several genes displayed distinct structural patterns compared with other members in the same subgroup. For example, *CmIAA*10 in Group Ⅵ and *CmIAA*5 in Group Ⅱ showed different exon-intron organisations from their corresponding subgroup members. Overall, *CmIAA* genes exhibited both conserved structural features and variations among different phylogenetic groups.

### Gene duplication patterns and collinearity analysis of the *CmIAA* family

**Fig. 4:**
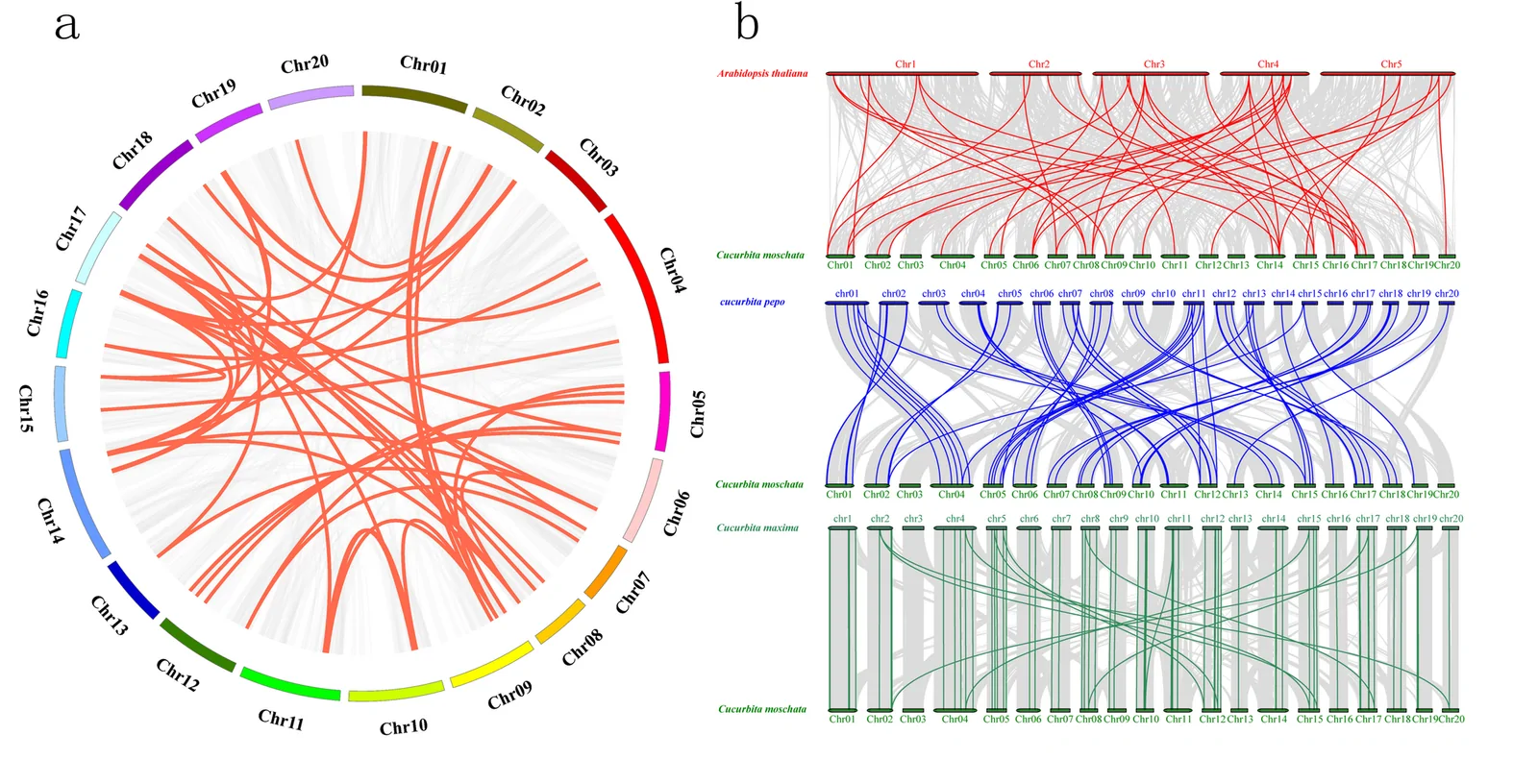
Synteny analysis of *CmIAA* genes within *C. moschata* and among different plant species. **a **represents intraspecies synteny of *CmIAA* genes in *C. moschata*, where the colored outer blocks indicate chromosomes and red lines denote segmentally duplicated *CmIAA* gene pairs. **b **show interspecies synteny of Aux/IAA genes between *C. moschata* and *Arabidopsis thaliana* (top), Cucurbita pepo (middle), and Cucurbita maxima (bottom), respectively. Colored lines indicate orthologous gene pairs

To investigate the evolutionary characteristics of the *CmIAA* gene family, intra- and interspecific collinearity analyses were performed (Fig. 4). Among the 72 identified *CmIAA* genes, 54 genes were involved in segmental duplication events, whereas no tandem duplication events were detected. These results indicate that segmental duplication contributed substantially to the expansion of the *CmIAA* gene family in pumpkin.

Intragenomic collinearity analysis revealed extensive syntenic relationships among *CmIAA* genes located on different chromosomes (Fig. 4a). The most prominent collinear relationships were observed between chromosomes 8 and 17, while several duplicated gene pairs were also distributed across chromosomes 1, 5, and 12. Furthermore, *Ka/Ks* analysis showed that the values of all duplicated gene pairs ranged from 0.0505 to 0.4093, with all ratios below 1. These results indicate that duplicated *CmIAA* genes have experienced predominant purifying selection during evolution.

Interspecific collinearity analysis was further conducted between pumpkin and three representative plant species, including *Arabidopsis thaliana*, *Cucurbita pepo*, and *Cucurbita maxima* (Fig. 4b). A total of 49, 64, and 77 orthologous gene pairs were identified between pumpkin and A. *thaliana*, C. *pepo*, and C. *maxima*, respectively. The highest number of syntenic gene pairs was observed between pumpkin and C. *pepo*, including both one-to-one and one-to-many orthologous relationships, consistent with their close evolutionary relationship within Cucurbitaceae.

### Promoter analysis of *CmIAA* genes

**Fig. 5:**
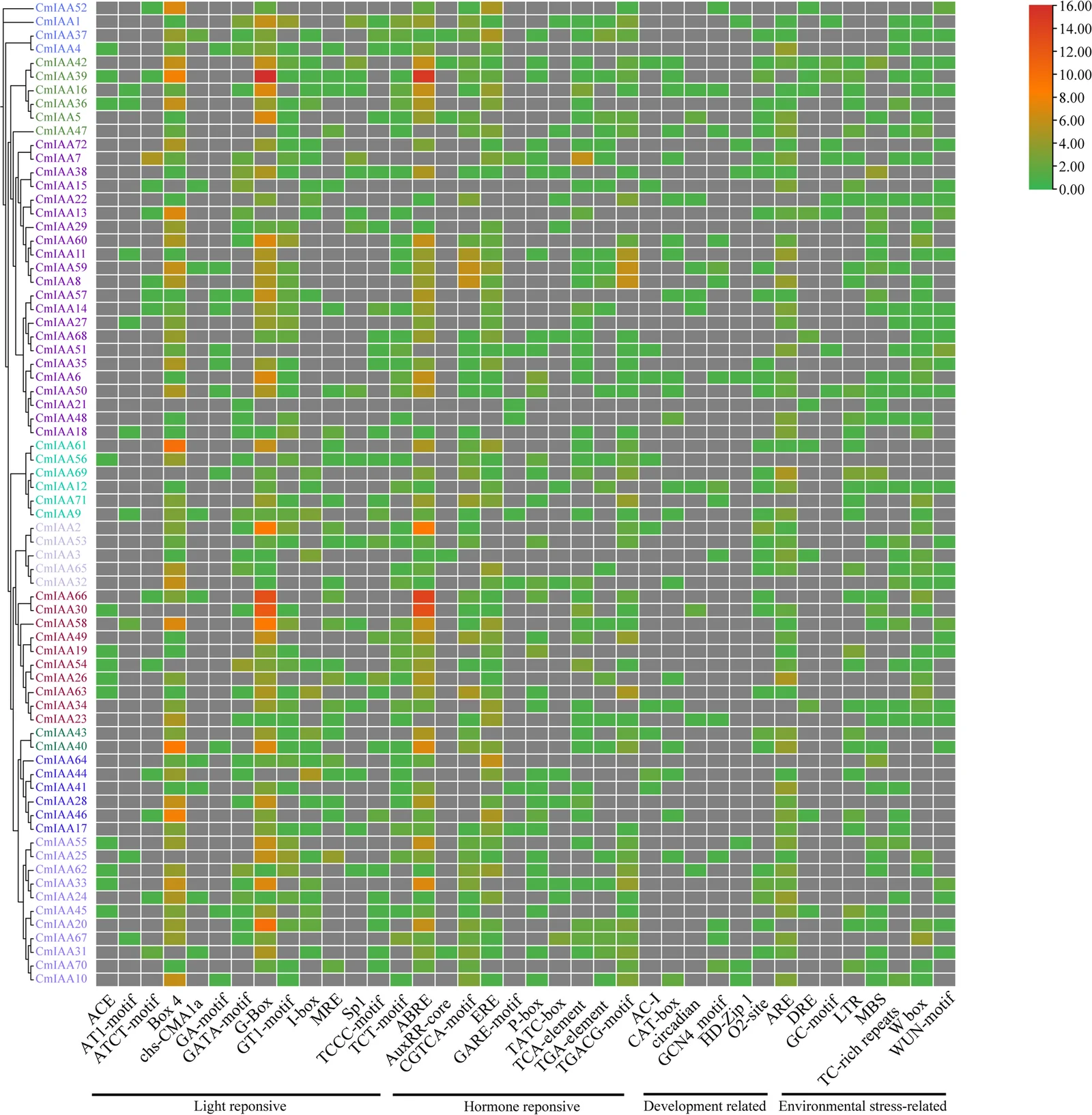
Cis-acting element analysis of *CmIAA* gene promoters. The heatmap shows the distribution and frequency of cis-acting regulatory elements identified in the 2 kb upstream promoter regions of *CmIAA* genes. The color scale represents the number of each cis-element detected in individual promoters

To investigate the potential regulatory characteristics of *CmIAA* genes, cis-acting elements within the 2 kb upstream promoter regions of all *CmIAA* genes were analyzed using the PlantCARE database (Fig. 5). The detailed numbers and distributions of identified cis-elements are provided in Additional file 2. A total of 38 types of cis-acting elements were detected and classified into four major categories, including light responsiveness, hormone responsiveness, developmental regulation, and environmental stress responsiveness.

Light-responsive elements were widely distributed among CmIAA promoters, indicating that light-related regulatory elements represent a common feature of the *CmIAA* gene family. Hormone-responsive elements were also frequently detected, including elements associated with auxin, abscisic acid, methyl jasmonate, and other hormone responses. In comparison, development-related elements showed relatively lower abundance among the analyzed promoters.

Several stress-responsive elements, including ABRE (abscisic acid-responsive element), MBS (MYB-binding site involved in drought inducibility), and DRE (dehydration-responsive element) were identified in the promoter regions of *CmIAA* genes. For example, *CmIAA*69 and *CmIAA*39 contained multiple stress-associated elements in their promoter regions. Combined with the subsequent expression analysis, several *CmIAA* genes exhibited differential transcriptional responses under salt and drought treatments. These results reveal diverse promoter architectures among *CmIAA* members and provide information for further investigation of their regulatory characteristics.

### Tissue-specific expression profiles of *CmIAA* genes

**Fig. 6:**
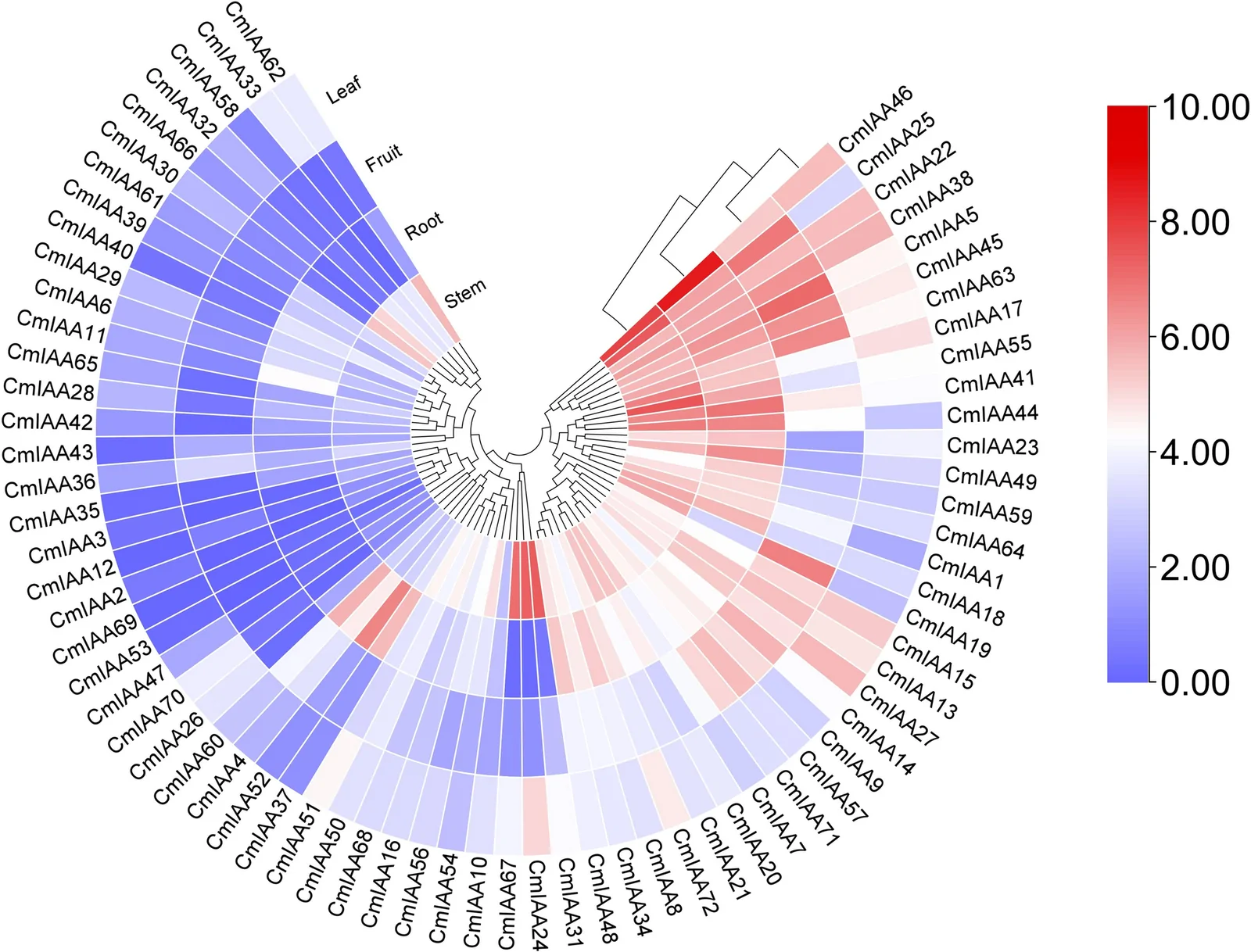
Tissue-specific expression profiles of *CmIAA* genes in pumpkin. The circular heatmap illustrates the expression patterns of *CmIAA* genes in root, stem, leaf, and fruit tissues based on RNA-seq data. transcript levels were normalized and log2-transformed, Significant differences among expression levels were determined at *P* < 0.05. Detailed statistical results and significance annotations are provided in Additional file 3

To characterize the expression patterns of *CmIAA* genes across different tissues, transcriptome data were used to analyze the expression levels of 72 *CmIAA* genes in roots, stems, leaves, and fruits (Fig. 6; Additional file 3). The expression profiles revealed distinct tissue-preferential patterns among *CmIAA* members. Several *CmIAA* genes exhibited relatively higher expression levels in roots, including *CmIAA*46, *CmIAA*41, *CmIAA*52, and *CmIAA*49. In stems, *CmIAA*24, *CmIAA*31, *CmIAA*55, and *CmIAA*67 showed relatively higher expression levels compared with other tissues. In contrast, many *CmIAA* genes displayed lower expression levels in leaves and fruits.

To validate the transcriptome-based expression patterns, qRT-PCR analysis was performed for all 72 *CmIAA* genes in roots and leaves (Additional file 4). The qRT-PCR results showed expression trends generally consistent with RNA-seq data, confirming the reliability of the transcriptomic expression analysis. Overall, *CmIAA* genes exhibited diverse tissue-specific expression patterns, with relatively higher expression levels observed in roots and stems than in leaves and fruits. These results demonstrate transcriptional differences among *CmIAA* members across pumpkin tissues.

### Expression profiles of *CmIAA* genes under abiotic stress conditions

**Fig. 7:**
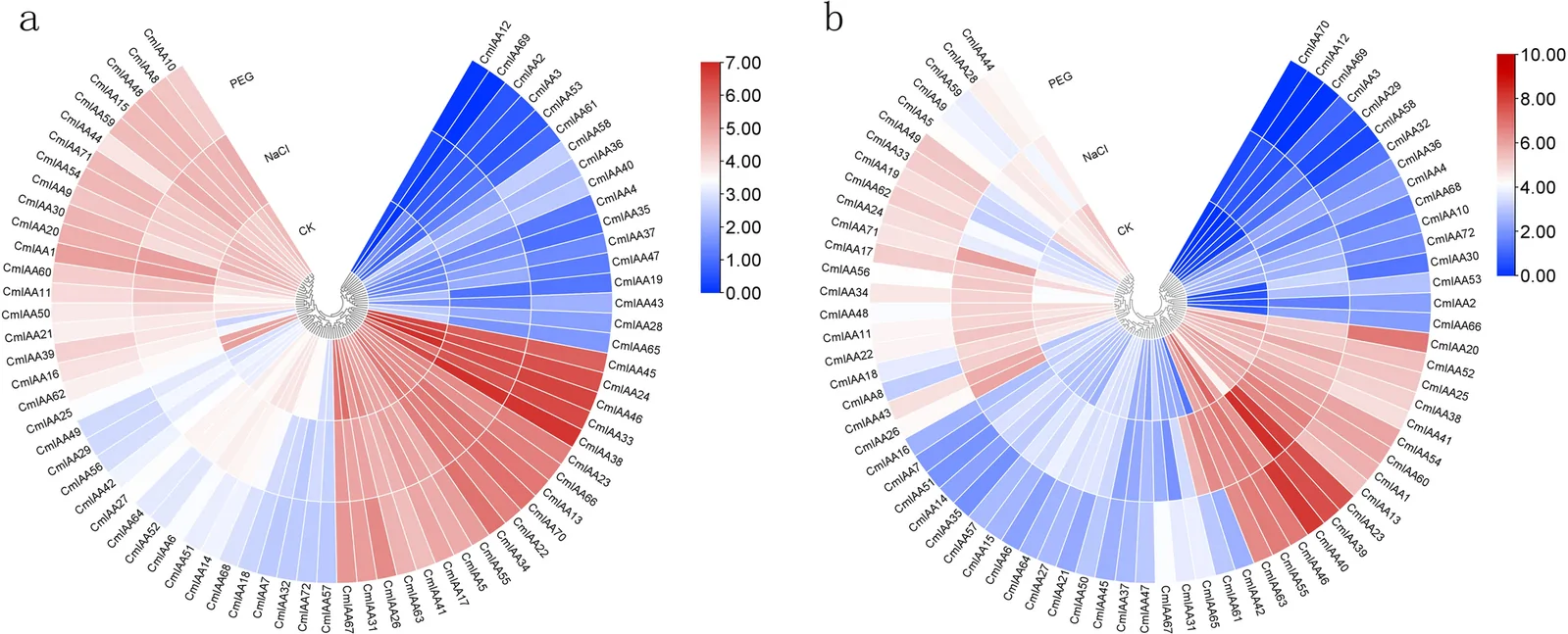
Expression patterns of *CmIAA* genes in leaves and roots under abiotic stress treatments. **a **Circular heatmap illustrating the expression profiles of *CmIAA* genes in leaf tissues under control (CK), salt (NaCl), and drought (PEG) treatments. **b **Circular heatmap illustrating the expression profiles of *CmIAA* genes in root tissues under the same abiotic stress treatments. Expression levels were normalized and log₂-transformed. Color gradients from blue to red indicate low to high expression levels, respectively. Significant differences among treatments were determined at *P* < 0.05. Detailed statistical results and significance annotations are provided in Additional file 5

To investigate the transcriptional responses of *CmIAA* genes to abiotic stresses, qRT-PCR analysis was performed to examine the expression patterns of 72 *CmIAA* genes in leaves and roots under salt (NaCl) and drought (PEG) treatments (Fig. 7; Additional file 5). Expression differences among treatments were statistically evaluated using one-way ANOVA followed by Tukey’s multiple comparison test, and significant differences were determined at *P* < 0.05. The detailed statistical results and significance annotations are provided in Additional file 5.

In leaves (Fig. 7a), several *CmIAA* genes displayed significant transcriptional changes under stress treatments. *CmIAA*39 was significantly upregulated under both NaCl and PEG treatments compared with the control, whereas *CmIAA*2 and *CmIAA*19 showed significant downregulation under both stress conditions. In roots (Fig. 7b), *CmIAA*58 and *CmIAA*66 were significantly induced by both NaCl and PEG treatments, with stronger expression increases observed under NaCl treatment.

Notably, *CmIAA*69 exhibited a salt-specific expression pattern in both leaves and roots, showing significant induction under NaCl treatment but no significant response under PEG treatment. In addition, *CmIAA*39 in leaves and *CmIAA*58 in roots showed different response patterns between salt and drought stresses, indicating tissue-dependent transcriptional responses among *CmIAA* members.

Overall, the qRT-PCR results revealed that different *CmIAA* genes exhibited distinct transcriptional responses under salt and drought stresses. Specifically, *CmIAA*39 responded to both salt and drought treatments, *CmIAA*58 was induced under both stresses with stronger activation under salt treatment, and *CmIAA*69 showed a salt-specific induction pattern. These results identify stress-responsive *CmIAA* candidates with distinct expression characteristics under different abiotic stress conditions.

## Discussion

### Evolutionary expansion and conservation of the *CmIAA* gene family

The *Aux/IAA* gene family constitutes a core regulatory component in the auxin signaling pathway and plays critical roles in plant growth, development, and responses to abiotic stresses [14, 34, 35]. In this study, a total of 72 *CmIAA* genes were identified in the pumpkin (*C. moschata*) genome. representing a relatively expanded *Aux/IAA* family compared with several previously characterized plant species, including *Arabidopsis thaliana* and *Oryza sativa*. The larger number of *CmIAA* members suggests that this gene family has undergone expansion during pumpkin evolution [36]. Collinearity analysis indicated that segmental duplication contributed substantially to the expansion of the CmIAA family, which is consistent with findings reported in *Solanum lycopersicum*, C*ucumis sativus*, and Brassicaceae species [13, 37].

The uneven distribution of *CmIAA* genes among chromosomes and phylogenetic subgroups further indicates that *Aux/IAA* family expansion was not uniform during evolution, a phenomenon that has also been observed in S*olanum lycopersicum* and *Brassica rapa* [13, 38]. Phylogenetic analysis further revealed Groups I, VI, and IX containing relatively more *CmIAA* members. Comparative studies in closely related Cucurbitaceae species, including *Cucumis sativus* [39], *Cucumis melo* [40], and *Citrullus lanatus* [41], have reported differences in *Aux/IAA* family size among these species. Although subgroup classifications may vary slightly among studies due to differences in species selection and phylogenetic analysis methods, the relatively larger number of *Aux/IAA* members identified in pumpkin suggests that certain *Aux/IAA* lineages may have experienced differential expansion or retention following duplication events. Such lineage-specific expansion may have increased the diversity of *Aux/IAA* members in pumpkin, providing potential resources for functional diversification within this gene family [42].

The *Ka/Ks* values of duplicated *CmIAA* gene pairs were consistently lower than 1, indicating that these genes have experienced strong purifying selection after duplication. In addition, the conserved syntenic relationships between pumpkin and other Cucurbitaceae species further support the evolutionary conservation of *Aux/IAA* genomic regions [43]. Together, these findings indicate that duplication events contributed to the expansion of the *CmIAA* family, while subsequent selective constraints maintained the conservation of Aux/IAA members during evolution [44].

### Gene structure and protein conservation

At the protein structural level, most CmIAA proteins retained the characteristic conserved motifs of the Aux/IAA family. particularly motif 1, indicating strong structural conservation within this gene family [35]. Similar conservation of Aux/IAA protein motifs has also been reported in other plant species, supporting the evolutionary stability of core Aux/IAA protein features.

Despite this overall conservation, differences in motif composition and exon–intron organization were observed among different phylogenetic groups, indicating structural diversification among CmIAA members. For example, *CmIAA*10 in Group Ⅵ contained an additional intron compared with other members of the same subgroup, representing a case of variation within a relatively conserved gene structure. Such structural differences may contribute to the diversification of CmIAA proteins during evolution [45].

Aux/IAA proteins contain several conserved domains that are essential for auxin signaling, including Domain Ⅱ, which mediates auxin-dependent protein degradation through the SCF^TIR1/AFB pathway [8, 46]. The conservation of these structural features among CmIAA proteins suggests that the fundamental mechanism of Aux/IAA-mediated auxin signaling has been maintained in pumpkin [47].

### Cis-regulatory elements of *CmIAA* genes in stress response

Cis-regulatory element analysis revealed that the promoter regions of *CmIAA* genes contain diverse regulatory elements associated with hormone responses, developmental regulation, and environmental stimuli, indicating transcriptional regulatory complexity and potential functional diversification within the *Aux/IAA* gene family [48]. The wide distribution and gene-specific composition of these cis-elements suggest that different *CmIAA* members may possess distinct transcriptional regulatory characteristics.

Several stress-associated cis-elements, including ABRE, MBS, and DRE, were identified in the promoters of *CmIAA* genes. The occurrence of these elements indicates that some *CmIAA* members contain promoter features related to abiotic stress responsiveness [49].Combined with the qRT-PCR results, several *CmIAA* genes showed differential transcriptional responses under salt and drought treatments, suggesting a potential relationship between promoter architecture and stress-responsive expression patterns.

Currently, Similar observations have been reported in other plant species. For example, in *Trifolium repens*, promoters of *TrIAA* genes were reported to contain stress-responsive elements, including MBS, and *TrIAA*6 and *TrIAA*7 exhibited inducible expression under salt stress [50]. Similarly, several *AoIAA* genes in *Asparagus officinalis* were found to contain drought-responsive MBS and anaerobic-responsive ARE elements in their promoter regions, indicating diverse regulatory characteristics of *Aux/IAA* members under environmental conditions [48].

Taken together, these findings suggest that stress-responsive cis-elements represent important regulatory features associated with the transcriptional regulation of *Aux/IAA* genes under abiotic stress conditions [51]. The diverse cis-element profiles of *CmIAA* promoters provide insights into the potential regulatory mechanisms underlying their transcriptional responses.

### Tissue-specific expression and developmental implications

Tissue-specific expression analysis revealed distinct spatial expression patterns of *CmIAA* genes across different organs. The differential expression profiles among roots, stems, leaves, and fruits indicate that individual *CmIAA* members possess diverse spatial expression characteristics. This expression pattern is consistent with previous studies in *Arabidopsis thaliana* and *Zea mays*, where some *Aux/IAA* members exhibit preferential expression in roots and are associated with root developmental processes [52]. In this study, several *CmIAA* genes, including *CmIAA*46 and *CmIAA*55, showed relatively higher transcript abundance in roots and stems, suggesting that these members may be associated with tissue-specific auxin-related regulatory processes. Similar expression characteristics of *Aux/IAA* genes have also been observed in *Oryza sativa* [53] and *Triticum aestivum* [54].

In contrast, many *CmIAA* genes showed relatively lower expression levels in leaves and fruits, suggesting that their transcription may be regulated in a tissue- or condition-dependent manner. Comparable expression patterns have also been observed in *Solanum lycopersicum* and *Capsicum annuum*, supporting the diverse expression characteristics of *Aux/IAA* family members across plant species [19, 20]. Overall, the tissue-specific expression profiles of *CmIAA* genes provide preliminary insights into their potential involvement in different biological processes. However, the specific functions of individual *CmIAA* members require further functional validation.

### Stress-responsive expression and functional divergence

*Aux/IAA* gene family are early auxin-responsive transcriptional repressors that function as important components of the auxin signaling pathway [55]. To date, increasing evidence indicates that *Aux/IAA* genes are involved in plant responses to various abiotic stresses [15]. For example, in *Oryza sativa*, *OsIAA20* enhances drought and salt tolerance in transgenic plants by increasing proline and chlorophyll contents via the ABA signaling pathway [56]. Similarly, *OsIAA6* is strongly induced by drought stress, and its overexpression significantly improves drought tolerance in plants [53]. In *Arabidopsis thaliana*, loss-of-function mutations in *AtIAA*5, *AtIAA*6, and *AtIAA*19 affect drought-associated physiological processes through regulation of glucosinolate metabolism [57]. In addition, differential expression patterns of *Aux/IAA* genes under salt and drought stresses have been reported in *Capsicum annuum* [19], *Artemisia argyi* [58] and *Medicago sativa* [55], suggesting that *Aux/IAA* members exhibit diverse stress-responsive characteristics across plant species.

In the present study, qRT-PCR analysis revealed that multiple *CmIAA* genes displayed differential expression patterns under salt and drought treatments, indicating diverse transcriptional responses among *CmIAA* members. The variation in stress-responsive expression patterns may be related to differences in promoter regulatory composition. [59]. For example. *CmIAA*69 showed salt stress-specific responses, and its promoter contains abundant stress-related elements (3 ABRE, 3 MBS, and 5 ARE). In contrast, *CmIAA*39 and *CmIAA*58 were both upregulated under salt and drought stress, and their promoters were enriched in ABRE elements (15 and 6, respectively), with the promoter of *CmIAA*58 also containing 1 MBS and 1 ARE. The abundance of ABRE elements suggests strong ABA-dependent regulatory potential [44, 60]. The consistency between expression profiles and cis-element composition indicates that promoter regulatory features may contribute to the stress-responsive expression patterns of *CmIAA* genes in pumpkin [61]. However, the underlying regulatory mechanisms remain to be further elucidated.

## Conclusion

In this study, a total of 72 *CmIAA* genes were identified from the pumpkin (*C. moschata*) genome and systematically characterized. These genes are unevenly distributed across 20 chromosomes and were classified into nine phylogenetic groups. Comparative analysis of conserved motifs, gene structures, and protein domains revealed both structural conservation and diversification among CmIAA members. Segmental duplication was identified as the major contributor to *CmIAA* family expansion, with 54 duplicated gene pairs detected. Promoter cis-acting element analysis suggested that *CmIAA* genes contained diverse regulatory elements associated with light responsiveness, hormone responses, and abiotic stress responses, including ABRE, MBS, and DRE motifs. Furthermore, transcriptome and qRT-PCR analyses demonstrated tissue-specific expression patterns and identified several stress-responsive *CmIAA* genes, including *CmIAA*39, *CmIAA*58, and *CmIAA*69, which showed differential expression patterns under salt and drought treatments. Overall, this study provides a foundation for future functional studies of the pumpkin *Aux/IAA* gene family and offers valuable references for subsequent functional studies and molecular breeding of stress-resilient pumpkin.

## Materials and methods

### Plant materials and treatments

oThe plant material used in this study was the pumpkin (*Cucurbita moschata*) inbred line ‘360-3’, which was provided by the Pumpkin Germplasm Resources Innovation and Utilization Team of the College of Horticulture and Landscape Architecture, Henan Institute of Science and Technology. The seedlings were grown in a nursery under supplemental LED lighting with a temperature of 25/20 ℃(day/night), a 16 h light/8 h dark photoperiod, a relative humidity of 70–80%, and a light intensity of 150–200 µmol·m⁻²·s⁻¹‌. Upon full cotyledon expansion, the seedlings were hydroponically cultured in ½ Hoagland’s nutrient solution for two weeks. Following a 24h acclimation in distilled water, uniform-sized seedlings with consistent growth performance were subjected to drought and salt stress treatments, respectively. Specifically, drought stress was simulated by exposure to a 20% polyethylene glycol 6000 (PEG6000) solution prepared with distilled water for 3 h, whereas salt stress was imposed using a 200 mM NaCl solution prepared with distilled water for 3 h [62, 63]. Seedlings treated with distilled water alone served as the control. The experiment was conducted using a randomized block design, with three biological replicates for each treatment and six seedlings per replicate. After stress treatments, root and leaf tissues were immediately harvested for total RNA extraction and subsequent qRT-PCR analysis.

### Identification of *CmIAA* family members

Aux/IAA protein sequences from *Arabidopsis thaliana* and *Oryza sativa* were retrieved using TBtools and employed as query templates. Homology-based searches were conducted against the pumpkin genome in the Cucurbit Genomics Database (CuGenDB) (http://cucurbitgenomics.org/rnaseq/pu/25) [64] using BLAST. Redundant sequences were removed using a similarity threshold of 50% and an e-value cutoff of 1 × 10 [50]. All non-redundant candidate proteins were subsequently verified for the presence of conserved Aux/IAA domains using NCBI-CDD (https://www.ncbi.nlm.nih.gov/Structure/bwrpsb/bwrpsb.cgi*)* and SMART (http://smart.embl-heidelberg.de/) [65]. Additional filtering steps were applied to eliminate any remaining redundancy, resulting in the final set of CmIAA family members.

### Prediction of physicochemical properties of CmIAA proteins

The ExPASy ProtParam tool (http://web.expasy.org/protparam/) was used to predict the basic physicochemical characteristics of each CmIAA protein, including the number of amino acids, molecular weight, and theoretical isoelectric point (pI) [66].

### Phylogenetic tree construction

A phylogenetic tree was constructed using the neighbor-Joining method with 1,000 bootstrap replicates in MEGA-X [67], based on the amino acid sequences of the CmIAA proteins. The resulting tree was subsequently refined and visualized using the Interactive Tree of Life (iTOL) online platform (https://itol.embl.de/) [68].

### Collinearity analysis

Gene family files for *Cucurbita pepo*, *Cucurbita maxima*, and *Arabidopsis thaliana* were obtained from Ensembl Plants (https://plants.ensembl.org/index.html), and those for pumpkin were downloaded from CuGenDB [64]. Both interspecific synteny analysis among these species and intraspecific synteny analysis of pumpkin *CmIAA* genes were conducted via TBtools [69]. Furthermore, to assess selection pressure, Ka/Ks ratios between duplicated *CmIAA* gene pairs were determined using KaKs_Calculator 2.0 [36].

### Promoter Cis-regulatory element analysis

Coding sequences (CDS) and full-length genomic sequences of all 72 *CmIAA* genes were retrieved from CuGenDB [64]. A 2,000 bp upstream region from each gene was extracted using TBtools (https://github.com/CJ-Chen/TBTools) and analyzed for cis-regulatory elements using the PlantCARE (http://bioinformatics.psb.ugent.be/webtools/plantcare/html/) [70] database. The results were graphically visualized with TBtools [69].

### Chromosomal localization

The chromosomal positions of all 72 *CmIAA* genes were obtained from CuGenDB [64]. Their distribution across chromosomes was mapped and visualized using TBtools [69].

### Gene structure and conserved motif analysis

Conserved motifs within the CmIAA protein sequences were identified using the MEME online program (https://meme-suite.org/meme/doc/meme.html). Gene structure information for all CmIAA members was obtained using the Gene Structure Display Server (GSDS 2.0) web server (https://gsds.gao-lab.org/Gsds_help.php) [71].

### Tissue expression pattern analysis

RNA-seq datasets of four tissue types (root, stem, leaf, and fruit) in pumpkin were downloaded from the CuGenDB database (https://www.ncbi.nlm.nih.gov/bioproject/PRJNA385310/) (PRJNA385310) [64]. Fragments per kilobase of transcript per million mapped reads (FPKM) values were calculated to estimate the transcript levels of CmIAA genes. The expression data were log2 transformed, and heatmaps were constructed using TBtools [40] to visualize the expression patterns across different tissues.

### RNA extraction and qRT-PCR analysis of abiotic stress treatment

Expression profiles of *CmIAA* genes under abiotic stress conditions were analyzed using quantitative real-time PCR (qRT-PCR) [50]. Briefly, total RNA was extracted from collected samples using the Omni Plant RNA Kit (CWBIO, Beijing, China) according to the manufacturer’s instructions. First-strand cDNA synthesis and qRT-PCR amplification were performed using commercial reagent kits purchased from TaKaRa (Dalian, China). All reactions were conducted on a Bio-Rad real-time fluorescence quantitative PCR system. The qRT-PCR reaction mixture (10 µL) consisted of 5 µL TB Green^®^ Premix Ex Taq II (TaKaRa, Dalian, China), 0.4 µL each of the forward and reverse primers, 0.2 µL of cDNA template, and 4 µL of ddH_2_O. The thermal cycling conditions were as follows: an initial denaturation at 95 °C for 30 s, followed by 40 cycles of 95 °C for 15 s and 60 °C for 30 s. A melting curve program (95 °C for 5 s, 65 °C for 5 s, and 95 °C for 5 s) was performed to verify the specificity of the amplification products. The pumpkin β-*Actin* gene was utilized as an internal reference [72, 73], and the relative expression levels were quantified using the 2^−*∆∆Ct*^ method [74]. For qRT-PCR analysis, three independent biological replicates were performed, and each sample was analyzed with three technical replicates. Primers used for qRT-PCR are listed in Additional file 1.

### Data and statistical analysis

Microsoft Excel 2021 was used for data processing and visualization, and statistical analyses were performed using SPSS 22 software. Significant differences among treatment groups were determined using one-way analysis of variance (ANOVA) followed by Tukey’s multiple comparison test at P < 0.05.

## Supplementary Information

Additional file 1: List of primers for qRT-PCR in this study.

Additional file 2: Cis-acting element analysis of *CmIAA* gene promoters.

Additional file 3: FPKM values of *C. moschata* *Aux/IAA* genes in four major tissues.

Additional file 4: Validation of RNA-seq expression profiles of *CmIAA* genes by qRT-PCR in pumpkin leaves and roots.

Additional file 5: Expression profiles of *C. moschata Aux*/*IAA* genes in response to salt and drought stress.

## Data Availability

No datasets were generated or analysed during the current study.

## References

[1] Xu H, Liu Y, Zhang S, Shui D, Xia Z, Sun J. Genome-wide identification and expression analysis of the AUX/IAA gene family in turnip (*Brassica rapa ssp. rapa*). BMC Plant Biol. 2023;23:342. 10.1186/s12870-023-04356-y PMC10294438 37370022

[2] Su Y, He H, Wang P, Ma Z, Mao J, Chen B. Genome-wide characterization and expression analyses of the auxin/indole-3-acetic acid (Aux/IAA) gene family in apple (*Malus domestica*). Gene. 2021;768:145302. 10.1016/j.gene.2020.145302 33181252

[3] Li C, Zameer R, Liu L, Wen Q, Zheng Y, Zheng J, et al. Harnessing the acid growth theory to optimize apoplastic acidification for enhancing cotton fiber elongation. Plant Commun. 2025;6:101390. 10.1016/j.xplc.2025.101390 PMC12281276 40443037

[4] Martin-Arevalillo R, Vernoux T. Decoding the auxin matrix: auxin biology through the eye of the computer. Annu Rev Plant Biol. 2023;74:387–413. 10.1146/annurev-arplant-102720-033523 36608348

[5] Leyser O. Auxin signaling. Plant Physiol. 2017;176:465–79. 10.1104/pp.17.00765 PMC5761761 28818861

[6] Bao D, Chang S, Li X, Qi Y, Crop. J. 2024;12:964–78.

[7] Luo J, Zhou JJ, Zhang JZ. Aux/IAA gene family in plants: molecular structure, regulation, and function. Int J Mol Sci. 2018;19:259. 10.3390/ijms19010259 PMC5796205 29337875

[8] Calderón Villalobos LIA, Lee S, De Oliveira C, Ivetac A, Brandt W, Armitage L, et al. A combinatorial TIR1/AFB–Aux/IAA co-receptor system for differential sensing of auxin. Nat Chem Biol. 2012;8:477–85. 10.1038/nchembio.926 PMC3331960 22466420

[9] Tan X, Calderon-Villalobos LIA, Sharon M, Zheng C, Robinson CV, Estelle M, et al. Mechanism of auxin perception by the TIR1 ubiquitin ligase. Nature. 2007;446:640–5. 10.1038/nature05731 17410169

[10] Li H, Wang B, Zhang Q, Wang J, King GJ, Liu K. Genome-wide analysis of the auxin/indoleacetic acid (Aux/IAA) gene family in allotetraploid rapeseed (*Brassica napus* L). BMC Plant Biol. 2017;17:204. 10.1186/s12870-017-1165-5 PMC5691854 29145811

[11] Shi Q, Zhang Y, To VT, Shi J, Zhang D, Cai W. Genome-wide characterization and expression analyses of the auxin/indole-3-acetic acid (Aux/IAA) gene family in barley (*Hordeum vulgare* L). Sci Rep. 2020;10:10242. 10.1038/s41598-020-66860-7 PMC7314776 32581321

[12] Jain M, Kaur N, Garg R, Thakur JK, Tyagi AK, Khurana JP. Structure and expression analysis of early auxin-responsive Aux/IAA gene family in rice (*Oryza sativa*). Funct Integr Genomics. 2006;6:47–59. 10.1007/s10142-005-0005-0 16200395

[13] Wu J, Peng Z, Liu S, He Y, Cheng L, Kong F, et al. Genome-wide analysis of Aux/IAA gene family in *Solanaceae* species using tomato as a model. Mol Genet Genomics. 2012;287:295–311. 10.1007/s00438-012-0675-y 22314799

[14] Weijers D, Wagner D. Transcriptional responses to the auxin hormone. Annu Rev Plant Biol. 2016;67:539–74. 10.1146/annurev-arplant-043015-112122 26905654

[15] Jing H, Wilkinson EG, Sageman-Furnas K, Strader LC. Auxin and abiotic stress responses. J Exp Bot. 2023;74:7000–14. 10.1093/jxb/erad325 PMC10690732 37591508

[16] Kumar A, Partap M, Warghat AR. From growth to survival: Aux/IAA genes in plant development and stress management. Plant Sci. 2026;362:112750. 10.1016/j.plantsci.2025.112750 40925440

[17] Zhu JK. Abiotic stress signaling and responses in plants. Cell. 2016;167:313–24. 10.1016/j.cell.2016.08.029 PMC5104190 27716505

[18] Waadt R, Seller CA, Hsu PK, Takahashi Y, Munemasa S, Schroeder JI. Plant hormone regulation of abiotic stress responses. Nat Rev Mol Cell Biol. 2022;23:680–94. 10.1038/s41580-022-00479-6 PMC9592120 35513717

[19] Waseem M, Ahmad F, Habib S, Li Z. Genome-wide identification of the auxin/indole-3-acetic acid (Aux/IAA) gene family in pepper, its characterisation, and comprehensive expression profiling under environmental and phytohormones stress. Sci Rep. 2018;8:12008. 10.1038/s41598-018-30468-9 PMC6089902 30104758

[20] Audran-Delalande C, Bassa C, Mila I, Regad F, Zouine M, Bouzayen M. Genome-wide identification, functional analysis and expression profiling of the Aux/IAA gene family in tomato. Plant Cell Physiol. 2012;53:659–72. 10.1093/pcp/pcs022 22368074

[21] Wang F, Niu H, Xin D, Long Y, Wang G, Liu Z, et al. OsIAA18, an Aux/IAA transcription factor gene, is involved in salt and drought tolerance in rice. Front Plant Sci. 2021;12:738660. 10.3389/fpls.2021.738660 PMC8637529 34868122

[22] Li G, Ye YX, Ren XQ, Qi MY, Zhao HY, Zhou Q, et al. The rice *Aux/IAA* transcription factor gene OsIAA18 enhances salt and osmotic tolerance in Arabidopsis. Biol Plant. 2020;64:454–64.

[23] Zou C, Tan H, Huang K, Zhai R, Yang M, Huang A, et al. Physiological characteristic changes and transcriptome analysis of maize (*Zea mays* L.) roots under drought stress. Int J Genomics. 2024;2024:5681174. 10.1155/2024/5681174 PMC10807950 38269194

[24] Singh V, Gupta K, Singh S, Jain M, Garg R. Unravelling the molecular mechanism underlying drought stress response in chickpea via integrated multi-omics analysis. Front Plant Sci. 2023;14:1156606. 10.3389/fpls.2023.1156606 PMC10242046 37287713

[25] Leftley N, Banda J, Pandey B, Bennett M, Voß U. Uncovering how auxin optimizes root systems architecture in response to environmental stresses. Cold Spring Harb Perspect Biol. 2021;13:a039917. 10.1101/cshperspect.a040014 PMC8559545 33903159

[26] Kim H, Subhin S, Youngdae Y, Geupil J. Jasmonic acid in plant abiotic stress tolerance and interaction with abscisic acid. Agronomy. 2021;11:1886.

[27] Chen W, Zhang M, Chen T, Wang J, Zhang Q. Genome-wide identification of Aux/IAA gene family and their expression analysis in *Prunus mume*. Front Genet. 2022;13:1013822. 10.3389/fgene.2022.1013822 PMC9597081 36313426

[28] Lei B, Huang Y, Xie JJ, Liu ZX, Zhen A, Fan M, et al. Increased cucumber salt tolerance by grafting on pumpkin rootstock and after application of calcium. Biol Plant. 2014;58:179–84.

[29] Chen C, Yu W, Xu X, Wang Y, Wang B, Xu S, et al. Research advancements in salt tolerance of *Cucurbitaceae*: from salt response to molecular mechanisms. Int J Mol Sci. 2024;25:9051. 10.3390/ijms25169051 PMC11354715 39201741

[30] Kulczyński B, Gramza-Michałowska A. The profile of carotenoids and other bioactive molecules in various pumpkin fruits (*Cucurbita maxima* Duchesne) cultivars. Molecules. 2019;24:3212. 10.3390/molecules24183212 PMC6766813 31487816

[31] Ali IH, Gülsah B. Effect of different salinity on seed germination, growth parameters and biochemical contents of pumpkin (*Cucurbita pepo* L.) seeds cultivars. Sci Rep. 2024;14:6929. 10.1038/s41598-024-55325-w PMC10960046 38519514

[32] Imani Asl E, Soorni A, Mehrabi R. Genome-wide characterization, functional analysis, and expression profiling of the Aux/IAA gene family in spinach. BMC Genomics. 2024;25:567. 10.1186/s12864-024-10467-z PMC11155116 38840073

[33] Gao J, Cao X, Shi S, Ma Y, Wang K, Liu S, et al. Genome-wide survey of Aux/IAA gene family members in potato (Solanum tuberosum): Identification, expression analysis, and evaluation of their roles in tuber development. Biochem Biophys Res Commun. 2016;471:320–7. 10.1016/j.bbrc.2016.02.013 26869512

[34] Zhuang Z, Bian J, Ren Z, Ta W, Peng Y. Plant Aux/IAA gene family: significance in growth, development and stress responses. Agronomy. 2025;15:1228.

[35] Li Y, Shaqila H, Yanhua Q. Advances in structure and function of auxin response factor in plants. J Integr Plant Biol. 2022;65:617–32. 10.1111/jipb.13392 36263892

[36] Fan S, Nan L, Huilong C, Zhu L, Chunjin L, Rong Z, et al. Large-scale analysis of the ARF and Aux/IAA gene families in 406 horticultural and other plants. Mol Hortic. 2024;4:13. 10.1186/s43897-024-00090-7 PMC11003162 38589963

[37] Gan D, Zhuang D, Ding F, Yu Z, Zhao Y. Identification and expression analysis of primary auxin-responsive Aux/IAA gene family in cucumber (*Cucumis sativus*). J Genet. 2013;92:513–21. 10.1007/s12041-013-0306-3 24371172

[38] Paul P, Dhandapani V, Rameneni JJ, Li X, Sivanandhan G, Choi SR, et al. Genome-wide analysis and characterization of Aux/IAA family genes in *Brassica rapa*. PLoS ONE. 2016;11:e0151522. 10.1371/journal.pone.0151522 PMC4822780 27049520

[39] Wu J, Liu S, Guan X, Chen L, He Y, Wang J, Lu G. Genome-wide identification and transcriptional profiling analysis of auxin response-related gene families in cucumber. BMC Res Notes. 2014;7:218. 10.1186/1756-0500-7-218 PMC4108051 24708619

[40] Ling Y, Chen X, Muhamatihan M, Wei Y, Yang S, Wang C, et al. Genome-wide identification and expression analysis of the melon (*Cucumis melo* L.) Aux/IAA gene family in response to abiotic and biotic stresses. Veg Res. 2026;6:e005.

[41] Hu Q, Yang J, Meng L, Liu J, Tian S. Genome-wide identification of Aux/IAA genes in watermelon reveals a crucial role for ClIAA16 during fruit ripening. Horticulturae. 2023;9:1167.

[42] Wang M, Feng G, Yang Z, Wu J, Liu B, Xu X, et al. Genome-wide characterization of the Aux/IAA gene family in orchardgrass and a functional analysis of DgIAA21 in responding to drought stress. Int J Mol Sci. 2023;24:16184. 10.3390/ijms242216184 PMC10671735 38003372

[43] Remington DL, Vision TJ, Guilfoyle TJ, Weller RJ. Contrasting modes of diversification in the Aux/IAA and ARF gene families. Plant Physiol. 2004;135:1738–52. 10.1104/pp.104.039669 PMC519086 15247399

[44] Cutler SR, Rodriguez PL, Finkelstein RR, Abrams SR. Abscisic acid: emergence of a core signaling network. Annu Rev Plant Biol. 2010;61:651–79. 10.1146/annurev-arplant-042809-112122 20192755

[45] Wu W, Liu Y, Wang Y, Li H, Liu J, Tan J, et al. Evolution analysis of the Aux/IAA gene family in plants shows dual origins and variable nuclear localization signals. Int J Mol Sci. 2017;18:2107. 10.3390/ijms18102107 PMC5666789 28991190

[46] Figueiredo MRAD, Strader LC. Intrinsic and extrinsic regulators of Aux/IAA protein degradation dynamics. Trends Biochem Sci. 2022;47:865–74. 10.1016/j.tibs.2022.06.004 PMC9464691 35817652

[47] Du W, Lu Y, Li Q, Luo S, Shen S, Li N, et al. TIR1/AFB proteins: active players in abiotic and biotic stress signaling. Front Plant Sci. 2022;13:1083409. 10.3389/fpls.2022.1083409 PMC9745157 36523629

[48] Wen S, Ying J, Ye Y, Cai Y, Li L, Qian R. Genome-wide identification and salt stress-responsive expression profiling of Aux/IAA gene family in *Asparagus officinalis*. BMC Plant Biol. 2025;25:759. 10.1186/s12870-025-06780-8 PMC12135281 40468198

[49] Wang H, Jones B, Li Z, Frasse P, Delalande C, Regad F, et al. The tomato Aux/IAA transcription factor IAA9 is involved in fruit development and leaf morphogenesis. Plant Cell. 2005;17:2676–92. 10.1105/tpc.105.033415 PMC1242265 16126837

[50] Qi T, Yang W, Hassan MJ, Liu J, Yang Y, Zhou Q, et al. Genome-wide identification of Aux/IAA gene family in white clover (*Trifolium repens* L.) and functional verification of TrIAA18 under different abiotic stress. BMC Plant Biol. 2024;24:346. 10.1186/s12870-024-05034-3 PMC11057079 38684940

[51] Shahid A, Wang W, Zhang Z, Xu L, De Bruyn R, Khan N. Genome-wide identification, expression and interaction analysis of ARF and AUX/IAA gene family in soybean. Front Biosci (Landmark Ed). 2022;27:251. 10.31083/j.fbl2708251 36042185

[52] Von Behrens I, Komatsu M, Zhang Y, Berendzen KW, Niu X, Sakai H, et al. Rootless with undetectable meristem 1 encodes a monocot-specific AUX/IAA protein that controls embryonic seminal and post-embryonic lateral root initiation in maize. Plant J. 2011;66:341–53. 10.1111/j.1365-313X.2011.04495.x 21219511

[53] Jung H, Lee DK, Choi YD, Kim JK. OsIAA6, a member of the rice Aux/IAA gene family, is involved in drought tolerance and tiller outgrowth. Plant Sci. 2015;236:304–12. 10.1016/j.plantsci.2015.04.018 26025543

[54] Qu L, Li Z, Zhang X, Zhang L, Liu X, Chen J, et al. Evolution of the Aux/IAA gene family in hexaploid wheat. J Mol Evol. 2017;85:107–19. 10.1007/s00239-017-9810-z 29085968

[55] Zhang J, Li S, Gao X, Liu Y, Fu B. Genome-wide identification and expression pattern analysis of the Aux/IAA (auxin/indole-3-acetic acid) gene family in alfalfa (*Medicago sativa*) and the potential functions under drought stress. BMC Genomics. 2024;25:382. 10.1186/s12864-024-10313-2 PMC11025244 38637768

[56] Zhang A, Yang X, Lu J, Song F, Sun J, Wang C, et al. OsIAA20, an Aux/IAA protein, mediates abiotic stress tolerance in rice through an ABA pathway. Plant Sci. 2021;308:110903. 10.1016/j.plantsci.2021.110903 34034863

[57] Salehin M, Li B, Tang M, Katz E, Song L, Ecker JR, et al. Auxin-sensitive Aux/IAA proteins mediate drought tolerance in *Arabidopsis* by regulating glucosinolate levels. Nat Commun. 2019;10:4021. 10.1038/s41467-019-12002-1 PMC6731224 31492889

[58] Lian C, Lan J, Ma R, Li J, Zhang F, Zhang B, et al. Genome-wide analysis of Aux/IAA gene family in *Artemisia argyi*: identification, phylogenetic analysis, and determination of response to various phytohormones. Plants (Basel). 2024;13:564. 10.3390/plants13050564 PMC10934841 38475411

[59] Huang X, Shad MA, Shu Y, Nong S, Li X, Wu S, et al. Genome-wide analysis of the auxin/Indoleacetic acid (Aux/IAA) gene family in autopolyploid sugarcane (*Saccharum spontaneum*). Int J Mol Sci. 2024;25:7473. 10.3390/ijms25137473 PMC11242263 39000581

[60] Ortiz-García P, González Ortega-Villaizán A, Onejeme FC, Müller M, Pollmann S. Do opposites attract? Auxin-abscisic acid crosstalk: new perspectives. Int J Mol Sci. 2023;24:3090. 10.3390/ijms24043090 PMC9960826 36834499

[61] Munns R, Millar AH. Seven plant capacities to adapt to abiotic stress. J Exp Bot. 2023;74:4308–23. 10.1093/jxb/erad179 PMC10433935 37220077

[62] Li H, Chen J, Zhao Q, Han Y, Li L, Sun C, et al. Basic leucine zipper (bZIP) transcription factor genes and their responses to drought stress in ginseng, *Panax ginseng* C.A. Meyer. BMC Genomics. 2021;22:316. 10.1186/s12864-021-07624-z PMC8088647 33932982

[63] Dudziak K, Zapalska M, Börner A, Szczerba H, Kowalczyk K, Nowak M. Analysis of wheat gene expression related to the oxidative stress response and signal transduction under short-term osmotic stress. Sci Rep. 2019;9:2743. 10.1038/s41598-019-39154-w PMC6391441 30808876

[64] Yu J, Wu S, Sun H, Wang X, Tang X, Guo S, et al. CuGenDBv2: an updated database for cucurbit genomics. Nucleic Acids Res. 2023;51:D1457–64. 10.1093/nar/gkac921 PMC9825510 36271794

[65] Letunic I, Khedkar S, Bork P. SMART: recent updates, new developments and status in 2020. Nucleic Acids Res. 2021;49:D458–60. 10.1093/nar/gkaa937 PMC7778883 33104802

[66] Gasteiger E, Hoogland C, Gattiker A, Duvaud SE, Wilkins MR, Appel RD, et al. Protein identification and analysis tools on the ExPASy server. In: Walker JM, editor. The Proteomics Protocols Handbook. Totowa: Humana; 2005. pp. 571–607.

[67] Kumar S, Stecher G, Li M, Knyaz C, Tamura K. MEGA X: molecular evolutionary genetics analysis across computing platforms. Mol Biol Evol. 2018;35:1547–9. 10.1093/molbev/msy096 PMC5967553 29722887

[68] Letunic I, Bork P. Interactive Tree of Life (iTOL) v6: recent updates to the phylogenetic tree display and annotation tool. Nucleic Acids Res. 2024;52:W78–82. 10.1093/nar/gkae268 PMC11223838 38613393

[69] Chen C, Wu Y, Li J, Wang X, Zeng Z, Xu J, et al. TBtools-II: a one for all, all for one bioinformatics platform for biological big-data mining. Mol Plant. 2023;16:1733–42. 10.1016/j.molp.2023.09.010 37740491

[70] Zhang J, Xu Y, Wu G, Niu C, Zhang Y, Hao J, et al. Genome-wide identification and expression analysis of the Aux/IAA gene family in lettuce (*Lactuca sativa* L). Int J Mol Sci. 2025;26:1687. 10.3390/ijms26041687 PMC11855171 40004151

[71] Jing X, Zou Q, Yang H. Genome-wide identification and characterization of the Aux/IAA gene family in strawberry species. Plants (Basel). 2024;13:2940. 10.3390/plants13202940 PMC11511250 39458886

[72] Miao L, Qin X, Gao L, Li Q, Li S, He C, et al. Selection of reference genes for quantitative real-time PCR analysis in cucumber (*Cucumis sativus* L.), pumpkin (*Cucurbita moschata* Duch.) and cucumber-pumpkin grafted plants. PeerJ. 2019;7:e6536. 10.7717/peerj.6536 PMC6475253 31024757

[73] Yuan J, Shen C, Chen B, Shen A, Li X. Genome-wide characterization and expression analysis of CAMTA gene family under salt stress in *Cucurbita moschata* and *Cucurbita maxima*. Front Genet. 2021;12:647339. 10.3389/fgene.2021.647339 PMC8249228 34220934

[74] Livak KJ, Schmittgen TD. Analysis of relative gene expression data using real-time quantitative PCR and the 2^–∆∆CT^ method. Methods. 2001;25:402–8. 10.1006/meth.2001.1262 11846609

